# Reduced Functional Connectivity in Adults with Persistent Post-Concussion Symptoms: A Functional Near-Infrared Spectroscopy Study

**DOI:** 10.1089/neu.2017.5365

**Published:** 2018-06-01

**Authors:** Lia M. Hocke, Chris C. Duszynski, Chantel T. Debert, Diane Dleikan, Jeff F. Dunn

**Affiliations:** ^1^ Hotchkiss Brain Institute, Calgary, Alberta, Canada.; ^2^ Department of Radiology, Experimental Imaging Lab, Calgary, Alberta, Canada.; ^3^ Department of Clinical Neurosciences, Foothills Medical Centre, Calgary, Alberta, Canada.; ^4^ Cumming School of Medicine Calgary, Alberta, Canada.

**Keywords:** concussion, DLPFC, fNIRS, functional connectivity, mTBI, working memory task

## Abstract

Concussion, or mild traumatic brain injury (mTBI), accounts for ∼80% of all TBIs across North America. The majority of mTBI patients recover within days to weeks; however, 14–36% of the time, acute mTBI symptoms persist for months or even years and develop into persistent post-concussion symptoms (PPCS). There is a need to find biomarkers in patients with PPCS, to improve prognostic ability and to provide insight into the pathophysiology underlying chronic symptoms. Recent research has pointed toward impaired network integrity and cortical communication as a biomarker. In this study we investigated functional near-infrared spectroscopy (fNIRS) as a technique to assess cortical communication deficits in adults with PPCS. Specifically, we aimed to identify cortical communication patterns in prefrontal and motor areas during rest and task, in adult patients with persistent symptoms. We found that (1) the PPCS group showed reduced connectivity compared with healthy controls, (2) increased symptom severity correlated with reduced coherence, and (3) connectivity differences were best distinguishable during task and in particular during the working memory task (n-back task) in the right and left dorsolateral prefrontal cortex (DLPFC). These data show that reduced brain communication may be associated with the pathophysiology of mTBI and that fNIRS, with a relatively simple acquisition paradigm, may provide a useful biomarker of this injury.

## Introduction

There are 1,600,000 mild traumatic brain injuries (mTBI) or concussion-related emergency department visits in the United States each year.^1^ Although the majority of mTBI patients recover within 1–3 months, 14–36% continue to have symptoms at 6 months, and up to 23% have been reported to stay symptomatic for several years.^2,3^ Conventional clinical imaging such as CT and MRI are insensitive to injury from mTBI.^4,5^ As a result, it is difficult to monitor injury progression or treatment response. There is a need for new imaging protocols, which could be used to further understanding of the pathophysiology of mTBI injury as well as provide a biomarker to aid clinicians in return to activity decisions.

This article reports on using functional near-infrared spectroscopy (fNIRS) to detect changes in regional brain communication as a marker of injury after mTBI. We define mTBI (in which we include concussion) as a functional injury caused by acceleration, deceleration, and rotational forces within the brain that result in metabolic disturbances and associated symptoms.^6^

fNIRS^7–11^ is a promising noninvasive optical imaging technique, which is sensitive to hemodynamic changes and has advantages for clinical settings because of its portability, ease of use, and cost efficiency.^12,13^ fNIRS uses NIR light through fiberoptics to transmit light into cortical tissue, in the spectrum where biological tissue is transparent and oxy- and deoxyhemoglobin are the main chromophores. The detected changes with fNIRS in oxy- and deoxyhemoglobin caused by neurovascular coupling are believed to indirectly reflect neuronal activity.^14,15^ fNIRS has been used to study regional brain communication by quantifying similarities in the oscillation frequencies of hemodynamic signals.^16^ This method is used to study resting-state networks in functional MRI (fMRI) by monitoring signal oscillations that are caused by changes in deoxyhemoglobin.^17,18^ Our group,^16,19,20^ and others,^21^ have used fNIRS to study hemodynamic oscillations using coherence analysis, which determines the degree of frequency-coupling between two signals, as a marker of regional communication

fMRI studies evaluating task-evoked changes in oxygenation, as well as functional connectivity, have shown potential for the objective assessment of mTBI.^22,23^ However, fMRI is only practical in large hospitals, clinics, and research settings because of the technologies' high cost and shielded environment. Therefore, an alternative tool to objectively measure and assess mTBI is needed. Previously, we used fNIRS to quantify interhemispheric communication (IHC) in pediatric patients with persistent post-concussion symptoms (PPCS).^16^ We found that IHC was reduced across the motor cortex in pediatrics with mTBI.

In this study, we expand on our earlier work by investigating fNIRS as a technique to assess cortical communication deficits in adults with PPCS. As symptoms involving working memory deficits are prominent in mTBI symptomology,^22,24^ we have added cognitive tasks to the acquisition protocol. The frontal cortex, and more specifically, the dorsolateral prefrontal cortex (DLPFC), is critically involved during working memory processes^25^ and, therefore, was included in this study. Lastly, for clinical purposes, it would be ideal to have a small hardware configuration with minimum fiber pairs to reduce cost, setup time, and transport issues. Therefore, we investigated the sensitivity to PPCS using a reduced number of fiber pairs in our study. This study supports that regional coherence is reduced during PPCS, and supports further investigation into the application of fNIRS as a tool for monitoring mTBI.

## Methods

### Subjects

The conjoint health ethics research board of the University of Calgary approved the research. Twelve mTBI patients with PPCS (5 males, 29 ± 10 years) (Table 1) were recruited from the Foothills Hospital Brain Injury Rehabilitation Clinic along with 12 healthy control participants (3 males, 30 ± 11 years), recruited through advertising and word of mouth. All subjects provided informed consent prior to participation in the study. Inclusion criteria for PPCS participants were (1) having been diagnosed with mTBI at least 3 months prior and currently experiencing persistent symptoms, (2) being between 18 and 50 years of age, (3) currently not on psychoactive drugs or medication (antidepressant, anxiety medication, recreational drugs), and (4) having no diagnosed neurological disorder. For controls, the same criteria (see criteria 2–4) were used. In addition, controls had no mTBI history, or had had only one mTBI, which had occurred >1 year prior to participation and resolved within the first few days.

**Table 1.** Demographics

*Condition*	*Age*	*Sex*	*Symptom score*	*Time post-injury (days)*	*Prior mTBI*	*Injury comment*
PPCS	22	F	41	385	3	Hit front of head on counter
PPCS	37	F	-	470	0	Hit back of head when fell
PPCS	20	F	70	290	0	Hit head above right eye when biking
PPCS	24	M	14	195	2	Hit head on running track - LOC
PPCS	38	M	13.5	365	5	Sports related - LOC
PPCS	18	F	24	435	1	Hit back of head during skiing
PPCS	18	M	34	210	10	Hit head on right side on glass, after body-check from the left
PPCS	41	F	14	305	3	Head was hit on the right side with rock
PPCS	32	M	91	273	30	Elbow below the nose
PPCS	18	M	31	152	3	Hit to the left side of cerebrum during hockey (fell head first)
PPCS	41	F	47	124	0	Hit bottom right jaw on end of waterslide; LOC for 1 min
PPCS	42	F	113	456	0	Hit head on steering wheel during car accident (whiplash)
Asympt.	25	M	0	255	3	Sports-related during football

Demographics of PPCS patients included in the study as well as one asymptomatic patient, with age (second column); sex (third column); symptom score (fourth column) from the Post-Concussion Symptom Inventory (PCSI) with additional Sport Concussion Assessment Tool 3 (SCAT3) questions; the time since injury until fNIRS measurement (fifth column); how many mild traumatic brain injuries (mTBIs) were diagnosed before this injury (sixth column) and how the injury was acquired (last column).

PPCS, persistent post-concussion symptoms; LOC, loss of consciousness; Asympt., The participant who was symptomatic when recruited, but asymptomatic when data acquired. Not included in the statistical analysis.

Originally 37 possible participants were recruited. Ten of the 37 participants were excluded because they failed to meet the inclusion criteria (severity of injury [*n* = 3], psychoactive medication [*n* = 5], and persistent symptoms [*n* = 2]). The latter two excluded participants were an acute mTBI patient, who had had an mTBI 5 days prior, and a patient (25-year-old male football player) who had been asymptomatic for 2 months after experiencing PPCS for 6 months. These participants were not included in the group-level analysis, but the asymptomatic patient was used in the correlation plot between severity and coherence values as additional comparison. The remaining three exclusions were because: the participant gave contradicting information (*n* = 1), the participant was colorblind (*n* = 1), and the data did not have adequate signal-to-noise ratio (SNR) in the majority of channels (*n* = 1). All participants provided demographic information and completed the Post-Concussion Symptom Inventory (PCSI) with additional Sport Concussion Assessment Tool 3 (SCAT3) questions, which are standard symptom measurement scales.

### NIRS

**FIG. 1.**
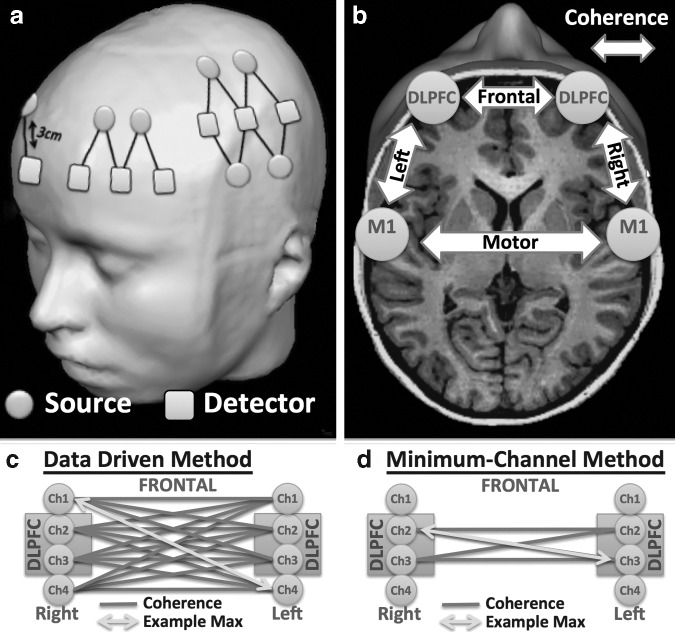
Setup and analysis. **(a)** Channel locations centered over the dorsolateral prefrontal cortex (DLPFC) (four channels) and primary motor cortex (M1) (eight channels). **(b)** Coherence was measured between (frontal, motor) and within hemispheres (right, left). (Modified picture from brainvoyager.com). Data were analyzed using two methods: **(c)** data driven method, in which all channels are used to find the maximal coherence between the two regions and **(d)** minimum-channel method, in which coherence between regions is calculated using only the two channels located directly above the DLPFC and M1 (10–20 system).

fNIRS measurements were continuously recorded with the TechEn CW7 using the wavelengths 690 and 830 nm at 50 Hz. We recorded four channels above and surrounding each right and left DLPFC and eight channels above and surrounding each left and right primary motor cortex (M1), respectively (Fig. 1a). The anatomical locations (DLPFC and M1) were measured and marked on the head based on the 10–20 electroencephalographic (EEG) coordinate system (for M1, the location was determined by taking 20% of the preauricular distance and applying this number from the vertex [Cz] to the left preauricular point; for the DLPFC we used the software Beam^26^). The four (over frontal cortex on each side) and eight channel probes (over motor cortex on each side) were centered over the anatomical locations, with the same two channels consistently positioned directly over the DLPFC and the M1 coordinate, respectively.

### Tasks

The participants were seated on a chair facing a computer screen in a dimly lit room, and were asked to perform several tasks. fNIRS data were collected continuously during the protocol. The tasks included: “Rest” (total, 180 sec), in which the participant was asked to relax and view a dark screen with a fixation point; “Tap,” for which the participant was asked to tap the right thumb sequentially to the other fingers in a self-paced rhythm (∼ 1 Hz; total time = 250 sec, 15 sec rest alternating with 10 sec tapping); “Stroop” task, for which the participant was presented with color words in a certain ink that alternated in blocks of unmatched (e.g., “red” in blue ink) and matched (e.g. “red” in red ink) words, for which the participant was asked to name the ink, not the written word (total time = 305 sec, 15 sec rest, with 15 sec task); “n-back,” for which position and letter had to be matched in a 0, 1, and 2-back paradigm.^27,28^ For the n-back task (total, 180 sec), 20 stimuli were presented in 2 sec intervals (40 sec) for each block (0, 1, and 2-back). The rest period between each block was 20 sec, plus an additional 5 sec of instructions prior to each block.

### Pre-processing

Raw fNIRS time courses were converted in HOMER2^29^ to oxy- (ΔHbO), deoxy- (ΔHb), and total-hemoglobin (ΔtHb) time courses. Preprocessing was done using MATLAB-based software packages (MATLAB 6.1, The MathWorks Inc., Natick, MA, 2000) and included motion correction with NAP^30^ and spline motion correction.^31^ In addition, individual channels were discarded when raw data were <90 dB (no signal) or >140 dB (oversaturated signal) as well as when SNR did not exceed SNR = 1. SNR was determined by the MATLAB function “snr” with unfiltered data as the input signal and high pass filtered data >1.5 Hz as the noise component. This calculation is similar to that of Sato and colleagues,^32^ arguing that <1.5 Hz, physiological signals such as cardiac, respiration, and low-frequency oscillations are expected, whereas above that cutoff, only noise is expected. Part of the low-frequency oscillations are believed to be an indirect measure of neuronal activity.^33^ This method achieved results identical to visual inspection of the noise level or the visual inspection of the presence of the cardiac waveform in the 830 nm raw data, which are common methods for channel exclusion.

### Post-processing

Preprocessed oxygenated fNIRS time courses (HbO) in the same hemispheres (e.g., between M1 and DLPFC centered channels) as well as between hemispheres (e.g., right with left DLPFC centered channels) were analyzed using coherence analysis^34^ at 0.04–0.1 Hz (Fig. 1b). The mean coherence in this frequency domain was calculated with static coherence, similar to our previous work.^16^

Cortical communication was determined by coherence, evaluating the “similarity” of two signals in the frequency domain. We used two methods to derive intra-hemispheric and inter-hemispheric coherence between and within the prefrontal and motor regions. First, for a general understanding of the sensitivity of inter-regional coherence, we used a “data driven” method (Fig. 1c), for which coherence was calculated among all channels in the four regions. In the data driven method, frontal channels include the channels directly over the right and left DLPFC, as well as all the neighboring channels for that region. The same is true for M1 and surrounding channels. For this method, the maximum coherence between each combination of regions was taken as the coherence value. Second, with the goal of simplifying the acquisition by reducing source-detector pairs to aid in future implementation in clinical settings, we investigated if similar sensitivity could be reached when we only analyzed two channels per region. We included in this “minimum-channel driven” method (Fig. 1d), the two channels directly above the DLPFC and M1 on the right and left side (see Methods section: NIRS) and calculated the maximum coherence between these channels. The reduction to two channels with the “minimum channel method” is to evaluate if the distinguishing location is confined to the DLPFC and M1 or is more diffuse in PPCS patients. In addition, fewer channels would make the method more clinically viable by significantly reducing the time to setup, and therefore make examinations quicker and more efficient. The reduction of needed channels also minimizes the size of the headgear needed, an important factor considering that mTBI patients commonly experience pressure in the head or headaches.

For each participant, four inter-regional coherence values per method were derived. Inter-regional coherence was quantified for (1) “frontal,” between the channels centered over the right and left DLPFC; (2) “motor,” between the channels centered over right and left M1; (3) “right,” between right DLPFC and right M1 centered channels; and (4) “left,” between the left DLPFC and left M1 centered channels.

### Statistical analysis

Statistical comparisons were done in SPSS (IBM corporation) using a mixed ANOVA with inter-regional coherence as the dependent variable, “PPCS” versus “Control” as the between-subject variable, and “Task” (four levels [e.g., n-back, Stroop]) and “Brain regions” (four levels [e.g., frontal, motor etc.]) as the within-subject variables. Post-hoc pairwise comparison was done with multiple two sample *t* tests (one sided, α = 0.05, adjusted for multiple comparison with the Benjamini–Hochberg method^35^). Correlation between severity (symptom scale) and overall coherence values were analyzed in MATLAB (function “corr”). The same function was used when evaluating the effect of the number of channels excluded in the analyses on the coherence values.

## Results

### NIRS parameters

**FIG. 2.**
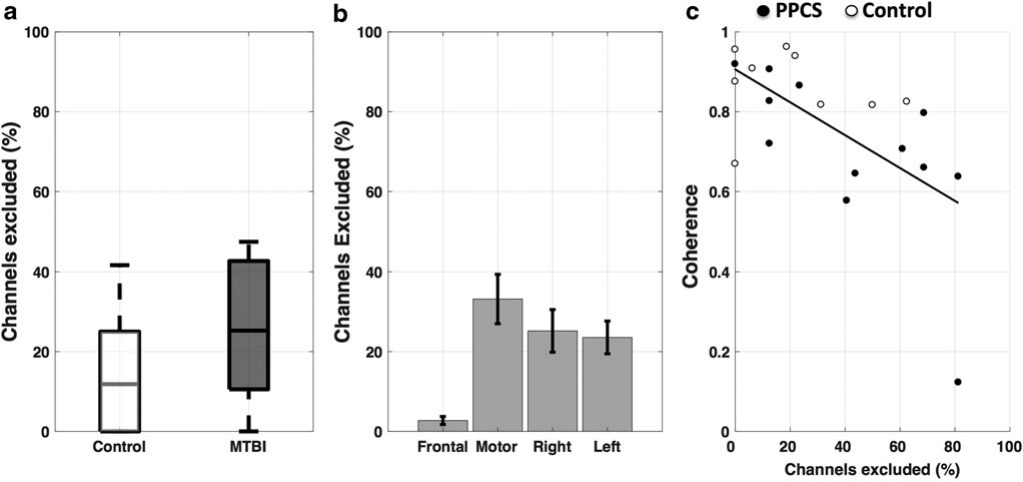
Evaluation of signal quality. The percent of channels that were excluded because of insufficient signal-to-noise ratio (SNR) **(a)** did not differ between the control and persistent post-concussion symptoms (PPCS) groups (mean ± SE), **(b)** differed between regions, and was highest in “motor” between left and right motor cortex, and **(c)** correlated strongly with coherence values in “motor” (*-r* = 0.62, *p* = 0.00) shown here, as well as “right” (*r* = −0.60, *p* = 0.00) not shown here.

As a first step, we evaluated the NIRS signal quality. For this study, as with any patient population, quick setup time is important to minimize discomfort and increase feasibility in clinical settings. However, quick setup can result in decreased signal quality if hair is not removed properly below the probes. For example, NIRS is sensitive to melanin in skin and hair, which can impede measurements. To evaluate the influence of poor signal quality on coherence values, we determined the number of channels, for each subject, which were below our criteria of quality (SNR <1) and compared the number of excluded channels with each coherence measure (Fig. 2). There was no significant difference between the “Control” and “PPCS” groups (*p* = 0.12) (Fig. 2a) in terms of percent of channels excluded. However, the number of channels (mean ± SD in percentage), which had insufficient SNR and were therefore excluded in our coherence calculations, differed for the regional combinations and were especially high for the “motor” 33 ± 28% and less so for the “right” 25 ± 25% and “left” 24 ± 20% (Fig. 2b). In the “frontal,” almost no channels had to be excluded 3 ± 5% (Fig. 2b). The number of channels we excluded for the regional combinations was significantly correlated with coherence values in the “motor” (-*r* = 0.62, *p* = 0.00) (Fig. 2c) “right” (*r* = −0.60, *p* = 0.00), but not in the “frontal” (*r* = −0.22, *p* = 0.31) and “left” (*r* = −0.39, *p* = 0.07).

In summary, the channels on the motor cortices had the highest amount of noise, having the lowest number of channels with sufficient SNR. The right motor cortex, in general, had fewer channels with sufficient SNR than the left side. In the frontal cortex (“frontal”), nearly all channels had consistently sufficient SNR across participants.

### Coherence

As previously noted, data were analyzed using two different methods: the “data driven method” and the “minimum channel method.” The focus of this article is to determine if we can distinguish PPCS patients and controls by measures of inter-regional coherence using multiple channels per region. For this reason, we focus on the results from the data driven method first, and discuss the “minimum channel method” later.

**FIG. 3.**
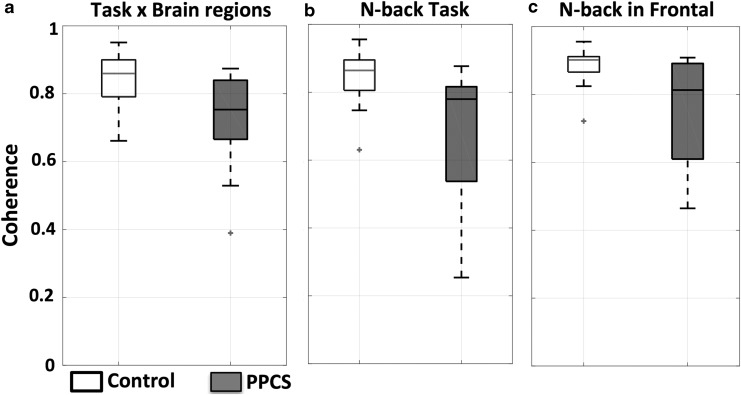
Reduced inter-regional communication in patients with persistent post-concussion symptoms (PPCS). **(a)** Visualization of difference in coherence between controls (white) and PPCS patients (black) across tasks and brain regions, **(b)** during n-back across brain regions, and **(c)** during n-back between left and right dorsolateral prefrontal cortex (DLPFC) centered channels (*p* = 0.05).

The following results apply to the data driven method (Fig. 1c) in which all fibers were used. We found a significant reduction in coherence in the PPCS group with a mixed ANOVA showing the fixed effect of the between-subject factor to be significant (*p* = 0.02, *F* = 6.48). Coherence values for the mTBI patients with PPCS and controls are shown as box plots for all regions and tasks combined in Figure 3a. The same is shown for the two groups during the n-back task for all regions (Fig. 3b), and only between the frontal regions “frontal” (Fig. 3c).

A different way to visualize the difference between the PPCS and control groups, as described in Figure 3, is presented in Table 2. The table shows how many PPCS patients (percentage), have coherence values below −2 SD of the control group mean. Discrimination between individuals with PPCS and controls was highest during the n-back task, in which 50% of the PPCS patients were below −2SD of the control group mean. Reduced coherence in patients with PPCS compared with control participants was significant (pairwise comparison–adjustment: Benjamini–Hochberg^35^) during the n-back “frontal” (50%, *p* = 0.05) and “left” (33%, *p* = 0.05) as well as during tap in “motor” (36%, *p* = 0.05) and during rest in the “right” (33%, *p* = 0.05) and the “motor” (33%, *p* = 0.05) (Table 2).

**Table 2.** Data Driven Method

	*Rest*	*Tap*	*Stroop*	*N-back*
Frontal	33%	8%	8%	^*^50%
Motor	^*^33%	^*^36%	9%	27%
Right	^*^33%	27%	9%	18%
Left	25%	8%	8%	^*^33%

Discrimination between persistent post-concussion symptom (PPCS) patients and controls separated by brain regions (rows) x tasks (columns). This table shows the percentage of PPCS patients who had coherence values below the mean − 2 SD of the controls. This table provides an overview of which combinations of task (columns) and connection (rows) are promising in distinguishing patients with PPCS from control coherence values on a more individual level.

^*^ Significant difference between the PPCS patients and controls based on independent sample *t* test (*p* = 0.05, adjusted for multiple comparison with the Benjamini–Hochberg method^35^).

Using the minimum-channel driven method (Fig. 1d), (where only two channels per region are used) pairwise comparison (adjustment, Benjamini–Hochberg^35^) showed significant differences between controls and patients with PPCS only during the n-back task in the “frontal” (58%, *p* = 0.04) (Table 3).

**Table 3.** Minimum-Channel Method

	*Rest*	*Tap*	*Stroop*	*N-back*
Frontal	42%	8%	8%	^*^58%
Motor	29%	14%	29%	0%
Right	43%	14%	14%	14%
Left	33%	11%	11%	22%

Discrimination between persistent post-concussion symptom (PPCS) patients and controls separated by brain regions (rows) x tasks (columns). This table shows the percentage of PPCS patients who had coherence values below the mean − 2SD of the controls. This table provides an overview of which combinations of task (columns) and connection (rows) are promising in distinguishing patients with PPCS from control coherence values on a more individual level.

^*^ Significant difference between the PPCS patients and controls based on independent sample *t* test (*p* = 0.05, adjusted for multiple comparison with the Benjamini–Hochberg method^35^).

**FIG. 4.**
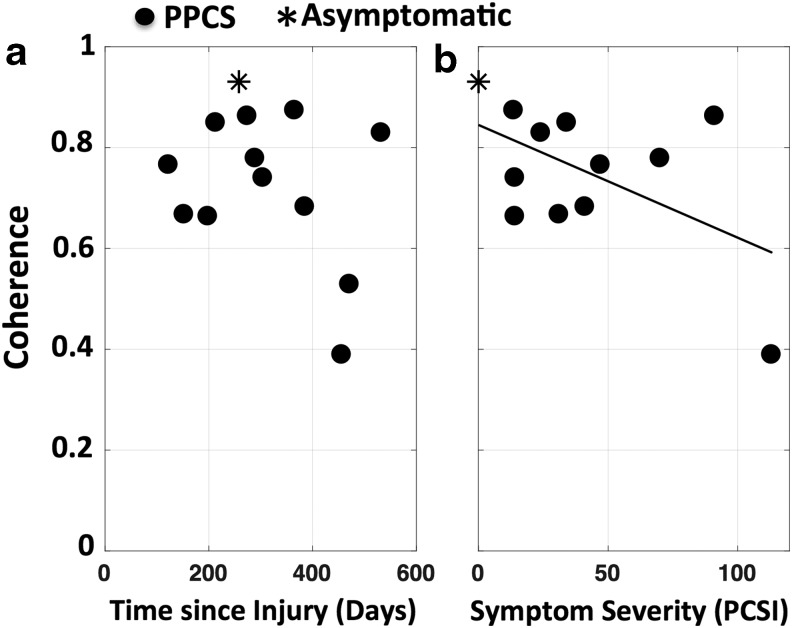
Inter-regional coherence decreases with severity of injury. Correlation between persistent post-concussion symptoms (PPCS) group (black dots) coherence values (across tasks and brain regions) and **(a)** time since injury, and **(b)** symptom severity (*r* = −0.53, *p* = 0.07). Black star represents a participant who was symptomatic when recruited, but asymptomatic when data were acquired.

Coherence values plotted against symptom severity are shown (Fig. 4) for all PPCS (black dots) patients, as well as for the patient who was recruited as symptomatic but reported no symptoms on the symptom inventory scales when the data were acquired (black star). Symptom severity measured with a combined PCSI and SCAT3 symptom score correlated highly with reduced coherence values, however it missed significance (*r* = −0.53, *p* = 0.07) (Fig. 4b). Coherence was not correlated with time since injury (*r* = −0.32, *p* = 0.28) (Fig. 4a).

In summary, we found significant discrimination between the control and PPCS group in various tasks and regional combinations, with coherence inversely correlated with the severity of the symptoms. High discrimination between the PPCS and control group, in which the coherence of patients with PPCS was significantly lower, was evident during the n-back task in regions that were not influenced by the number of channels with sufficient SNR. The minimum-channel method (Table 3) confirmed localized discrimination during the n-back task between the left and right DLPFC as seen in the data-driven method (Table 2).

## Discussion

We investigated whether we could distinguish healthy controls from mTBI patients with PPCS through measures of inter-regional coherence with fNIRS. Our results add to previous fMRI, and more recently added fNIRS studies of activation and connectivity alterations following mTBI. The focus on task-based network integrity, the range of tasks, and coverage of various distant brain areas, as well as our homogeneous sample of only chronic patients (>3 month post-injury), addresses several gaps in the literature and expands on our earlier pediatric study.^16^

### Reduced inter-regional coherence

Our results are comparable to our previous work on a pediatric population,^16^ showing a reduction in connectivity in patients with PPCS (Fig. 3). In addition, these results, along with our previous study, suggest that task-based connectivity provides higher discrimination for mTBI patients with PPCS than resting state connectivity. The n-back task was the best protocol, as we found that 50% (Table 2) of the PPCS patients' coherence values were below 2SD of the control mean. Further, the discrimination between patients and controls increased to 58% (Table 3) when analysis was restricted to channels directly over the DLPFC during the n-back task. These results are especially encouraging, as a localized DLPFC measurement (Table 3) would increase the speed and efficiency of setup, and exclude potentially compromised data from regions with lower SNR, such as the motor cortex (Fig. 2b,c).

Our data are supported by fMRI literature that shows a reduction of connectivity in mTBI patients. In most cases, functional connectivity studies of mTBI with MRI have been focused on resting-state measurements. Decreased resting state connectivity in mTBI patients was reported in the default mode network^36,37^ and for thalamofrontal connectivity^38^ in subacute mTBI patients as well as for decreased frontal connectivity in chronic mTBI patients.^39^ However, other studies have reported increases in frontal connections, including the prefrontal cortex.^36,40^ Only one study has examined task-based connectivity.^38^ Zhou and colleagues^38^ reported that during finger tapping, in contrast to rest, thalamomotor connectivity increased in controls but not in mTBI patients (3–58 days post-injury), supporting our task-based results. A review of these studies can be found in a number of studies.^37,41–43^ In summary, our study results are in agreement with the majority of the results from MRI connectivity studies in mTBI patients, which show a reduction of connectivity in frontal and motor regions.

There are several key mechanisms that might have contributed to the reduction in connectivity. The cascade of events that occur following mTBI include metabolic, hemodynamic, and structural changes.^44,45^ Axonal stretching and shearing occur with mTBI, causing diffuse axonal injury.^46–48^ The stretching and shearing can impair axonal transport and lead to reduced cortical communication. Such an injury can persist for months or even years.^49^ In addition, there is strong evidence for reductions in cerebral blood flow (CBF),^44^ which may last for months to years post-mTBI.^50^ fNIRS is sensitive to relative changes in hemoglobin that occur in response to neurovascular coupling, but does not provide a direct measurement of neuronal activation or CBF. Therefore, without further research, we cannot differentiate whether the alterations found in connectivity reflect dysfunction in metabolism, tract integrity, or blood flow regulation, all of which are known to affect cerebrovascular hemoglobin concentrations. In all, our findings of reduced coherence support fNIRS measurements as a promising biomarker to monitor mTBI patients.

### Network deficits during working memory

We found that reduced connectivity in patients with PPCS was especially evident during the working memory (n-back) task (Table 2) between prefrontal regions. The prefrontal regions were not influenced by SNR. Specifically, changes in connectivity during the n-back task were found to be localized to the DLPFC (Table 3). The prefrontal region and especially the DLPFC are key regions in working memory processing that are known to be active during n-back tasks (see meta-analysis^25^). Further, as mentioned previously, symptoms involving working memory deficits are prominent in mTBI symptomology,^22,24^ supporting our findings of reduced DLPFC connectivity during working memory tasks in the PPCS group.

fNIRS is a new technique with great potential to quantify hemodynamics in mTBI. We are aware of three studies that utilized fNIRS to investigate mTBI, all of which showed decreased magnitude of change in oxyhemoglobin with tasks in the left DLPFC. The changes were measured during a working memory task, after subacute (15–45 days)^51^ and chronic periods (1–23 months)^52,53^ post mTBI. Our results support these findings, showing altered oxygenation in the prefrontal areas during task (Table 2).

Our current results are also in line with most of the previous mTBI studies reporting differences in activation patterns using MRI in the frontoparietal regions, and more specifically, in the DLPFC during working memory tasks.^22,23^ Although these studies are solely focused on the magnitude of activation, they have shown that several factors influence brain activation in mTBI patients during n-back tasks. Two of these factors include (1) cognitive load of working memory task and (2) time post-injury.

Regarding the brain activation changes in mTBI patients with cognitive load during the n-back task, increased activation in the frontal cortex was found for mTBIs in the moderate cognitive-load; namely, 2-back >0-back contrast;^54,55^ and 2-back >1-back contrast.^27,56–58^ However, the lower cognitive load (0-back >1-back contrast) has been reported to lead to reduction in bilateral frontal and parietal regions in mTBI patients.^56^ In the current study, we showed a reduction in frontal connectivity during 0-, 1-, and 2-back combined, which is consistent with previously reported reduction of activation patterns in the frontal cortex.

It is likely that time post-injury is a critical factor when explaining possible differences between mTBI and controls.^22,23^ Many studies focus on acute to subacute symptomatic mTBI populations, from 3–12 days,^56^ 1 week,^55^ 72 h, 2 weeks,^27^ and 6–35 days post-injury.^56–58^ Only a few MRI studies have included chronic mTBI patients, and even fewer studies have focused on chronic patients with PPCS, as we did in the current study. One of these studies included mTBI patients with symptoms from 6 to 200 days^59^ and others included mTBI patients with symptoms persisting >1 month^60–62^ and 2 months.^63^ The studies found reduced bilateral DLPFC activation with fMRI,^60–63^ and reduced right^59^ and left^62^ DLPFC activation during working memory tasks, as well as no difference during an n-back task.^64^ These reductions in regional activation in chronic populations have been attributed to reduced blood flow, and are often accompanied by more widespread activation, believed to be a compensatory mechanism in these injured patients.^65^ We can only speculate whether diffuse compensatory task-activation or reduced blood flow, as previously documented in chronic mTBI patients with PPCS, could be the underlying cause of the reduced connectivity in the DLPFC that we found.

### Improvements to increase clinical applicability

fNIRS holds particular promise for clinical applications as it has few contraindications compared with other imaging modalities. fNIRS is portable, can be used in a natural environment (unlike shielded MRIs, or electrical interferences with EEG), with no contraindications (e.g., metal in MRI). It is even possible to collect data during activity (see NIRSport, made by NIRx Medical Technologies, LLC). For these reasons, fNIRS is suitable for bedside or even “on-the-field” (e.g., in sports) measurements.

In this study, we further demonstrate the potential clinical applicability of fNIRS, by reducing the channels needed for the discrimination between controls and mTBI patients (Table 3). By choosing anatomically relevant channel locations, such as the ones directly above M1 and the DLPFC, we were able to reduce the number of channels to well-defined brain areas. The ability to focus specifically on the two channels above the DLPFC (Table 3) is especially advantageous, as it reduces the setup time to mere minutes, and makes the method viable for clinical population without compromising on SNR.

### Limitations

We found that motor regions were highly influenced by SNR (Fig. 2). In the current study we used stringent preprocessing steps for excluding channels. The result is that more fiber pairs failed to meet our SNR requirements. This however, is a necessary step for the evaluation of connectivity through coherence, because we show that low SNR can lead to spurious coherence values because of a coherent lack of signal rather than the presence thereof. We showed that the number of usable channels influenced coherence values between the left and right motor cortices (“motor”) as well as between the right frontal cortex and the right motor cortex (“right”) (Fig. 2c). The higher density of hair over the motor cortex is the most likely cause of fewer channels meeting acceptance criteria. More work has to be done in acquisition hardware in fNIRS to reduce setup time without compromising on signal quality, which is especially necessary for patient populations with a low tolerance for long acquisition paradigms.

A general limitation of our study is that we did not control for various factors, which may have influenced brain activation patterns, such as sex, nutrition intake, time of day, or fatigue status. Nevertheless, population differences were detected.

## Conclusion

We show promising results supporting the capability of fNIRS coherence measures to evaluate reduced connectivity in mTBI patients with persistent symptoms. Reduced connectivity in mTBI patients with PPCS compared with controls was especially prominent during the working memory task between the right and left DLPFC. Further, we report that reduced connectivity in this population could be measured in easily accessible regions such as the DLPFC, with only two channels. Minimizing channels is beneficial in order to reduce cost, increase portability, and maximize ease of setup: improvements that increase the suitability of fNIRS for clinical application in studies of brain injury.

As stated, more research is needed to evaluate if fNIRS inter-regional coherence could be an objective aid for monitoring mTBI. A next step will be to measure connectivity in acute mTBI patients, and longitudinally follow these patients to determine if fNIRS connectivity measures can be predictive of outcomes following mTBI. fNIRS measures of coherence may provide a useful biomarker of injury and recovery, adding to our understanding of the pathophysiology of mTBI and providing a new method to assess treatment responses.
